# Taking titles and naming names - who do we really think we are?

**DOI:** 10.1186/1757-1146-8-S1-A3

**Published:** 2015-04-20

**Authors:** Dawn Bacon

**Affiliations:** 1 Professional Practice in Health Sciences Faculty, University of Southampton, Southampton, UK

## Aims

Derived from a larger exploration in podiatric specialisation, the research aims relevant to this presentation were to trace the evolution, change over time and impact of specialist titles.

## Content

Findings from extensive qualitative research employing a range of methodologies are presented. Content analysis elicited the range of podiatric specialist areas, when titles associated with specialisation entered the professional language of podiatry, how titles have been applied and changes to titles over time. Using diabetes as a clinical exemplar, findings from focus group and key actor interviews illustrate the impact of specialist titles. Via a criterion based, purposive sampling strategy informed respondents were accessed; in order to explore and build upon this baseline data, further informants were identified using snowball sampling. Respondents included podiatrists specialising in diabetes, diabetologists, NHS service managers and representatives from government and professional bodies. In addition to the research findings, social theories pertinent to the area of professional titles are briefly presented.

## Relevance

Research encompassing the origins and effects of professional titles in podiatry has not previously been presented and, as an area, remains under debated within the literature. Whether working in the NHS where titles were greatly influenced by the implementation of Agenda for Change, or in private practice, clinicians experience the effects of their titles and while some have strongly held views on their appropriateness, many remain ambivalent. The demonstrable impact of titles leads to consideration of their potential for the informed user, be they podiatrist or manager.

## Outcomes

Drawing on content analysis covering some sixty five years of job advertising in all of the main chiropody and podiatry journals, the presentation illustrates the emergence, changes over time and current uses of specialist titles in a range of areas. Against this background, titles assigned to podiatrists specialising in diabetes are explored. Findings from in depth key actor and focus group interviews elicit the impact of specialist titles, including changes to: accountability under the law, patients’ access to services, clinicians’ roles, how clinicians are perceived and services are designed.

## Discussion

The assignment of professional titles and how they are interpreted is moulded by historical, temporal, cultural and ideological influences. Within podiatry titles have evolved into more than simple descriptors; they have been used to convey concepts of differentiation, status and role. Titles have been employed both to mark new professional boundaries and to maintain existing demarcation. Their impact shapes the way in which practitioners, services and the profession are perceived, clinicians’ accountability under the law; and crucially they influence patients’ access to healthcare services. In the field of diabetes, podiatrists have been assigned numerous and varying titles, in particular use of the word “specialist” remains the subject of ongoing reflection and debate. Consideration of the wide reaching effects of professional titles, illuminated by social theory, remains an important area for podiatrists to address.

